# GLUT1 targeting and hypoxia-activating polymer-drug conjugate-based micelle for tumor chemo-thermal therapy 

**DOI:** 10.1080/10717544.2021.1992039

**Published:** 2021-10-20

**Authors:** Pengkai Ma, Guijie Wei, Jianhua Chen, Ziqi Jing, Xue Wang, Zhijun Wang

**Affiliations:** a School of Chinese Materia Medica, Beijing University of Chinese Medicine, Beijing, China; b Division of Interventional Radiology, Department of Geriatric Medicine & National Clinical Research Center of Geriatric Disease, the 2nd Medical Center of Chinese PLA General Hospital, Beijing, China; c Department of Interventional Radiology, the 1st&5th Medical Center of Chinese PLA General Hospital, Beijing, China

**Keywords:** Polyprodrug, micelle, mitochondria targeting, tumor microenvironment, chemo-thermal therapy

## Abstract

**Purpose:**

Mitochondria are closely correlated with the proliferation and metastasis of tumor for providing suitable micro-environment and energy supply. Herein, we construct a glucose transporter 1 (GLUT1) targeting and hypoxia activating polyprodrug-based micelle (Glu-PEG-Azo-IR808-S-S-PTX) for mitochondria-specific drug delivery and tumor chemo-thermal therapy.

**Results:**

The micelle was characterized by hypoxia-sensitive PEG outer layer detachment, high photo-thermal conversion efficiency, and glutathione (GSH)-sensitive paclitaxel （PTX） release. It showed GLUT1 specifically cellular uptake and hypoxia-sensitive mitochondria targeting on A549 cell. *In vivo* fluorescence imaging confirmed the micelle also could selectively accumulate in tumor and its mitochondria on A549 tumor-bearing nude mice. Consequently, it not only exhibited higher cytotoxicity, apoptosis rate, and metastasis inhibition rate on A549 cells, but also better tumor growth and metastasis inhibition rate on tumor-bearing nude mice and lower whole-body toxicity. The mechanism might be caused by destroying mitochondria and down-regulating ATP production.

**Conclusion:**

This study provided a GLUT1 targeting, hypoxia, and reductive responsive nanomedicine that hold the potential to be exploited for tumor therapy.

## 1. Introduction

Mitochondria are known as the powerhouse of the cell and the source of important mediators of apoptosis (Yamada et al., 2020). Recent studies discovered that it also played a central role in the occurrence and development of tumor metastasis. On one hand, the mitochondria produced excess superoxide free radical that initiated the formation of metastatic foci (Denisenko et al., 2019). On the other hand, excess lactic acid produced by glycolysis of mitochondria activated the proteolytic enzyme and angiogenesis that accelerated the degradation of extra-cellular matrix and paved escaping channel for metastasis (Deng et al., 2019; Gandhi & Das, 2019). Although mitochondria seem to be an appealing target for the development of anti-tumor metastasis pharmaceutics, this area is still far from being covered.

To specifically deliver drug to mitochondria, various nanocarriers such as liposomes (Yamada et al., 2007), nanoparticles (Gisbert-Garzaran et al., 2020), and PAMAM dendrimer (Ma et al., 2018) have been designed by the incorporation of mitochondria tropic agents. However, there exist some intrinsic limitations for these nanocarriers, such as low-drug loading, burst drug release, nanocarrier-associated toxicity, and poor stability (Ghosh et al., 2019). The polyprodrug-based micelle seems to be an alternative strategy to overcome these deficiencies (Lin et al., 2020; Liu et al., 2020a). When the functional groups of polymers conjugated with targeting ligand or therapeutic agents through stimuli-responsive linkers, the resulting amphiphilic polymeric prodrugs tend to self-assemble into micelles with tumor-targeting ability, high-drug loading, and low immunogenicity. Particularly, they are stable and inactive under normal conditions, but can realize controllable drug release, size shrink, or surface change when stimuli are applied (Deng & Liu, 2020).

The abnormal energy metabolism results in the over-expression of glucose transporter 1 (GLUT1) and glutathione (GSH) as well as lack of oxygen (Vander Heiden & DeBerardinis, 2017). Although pH, glutathione (GSH), and enzymes have been widely exploited to construct tumor microenvironment responsive drug delivery systems, the hypoxia is still far from being covered (Yin et al., 2020; Zhou et al., 2021). Moreover, the mitochondria are more susceptible to hyperthermia under hypoxic tumor microenvironment (TME). Interestingly, IR808, a photothermal agent, also exhibited excellent mitochondrial targeting ability and has been used for mitochondria chemo-photothermal therapy to conquer tumors in recent studies. Therefore, hypoxia-activated mitochondria targeting chemo-thermal therapy might be worth exploring for tumor therapy.

**Figure 1.**
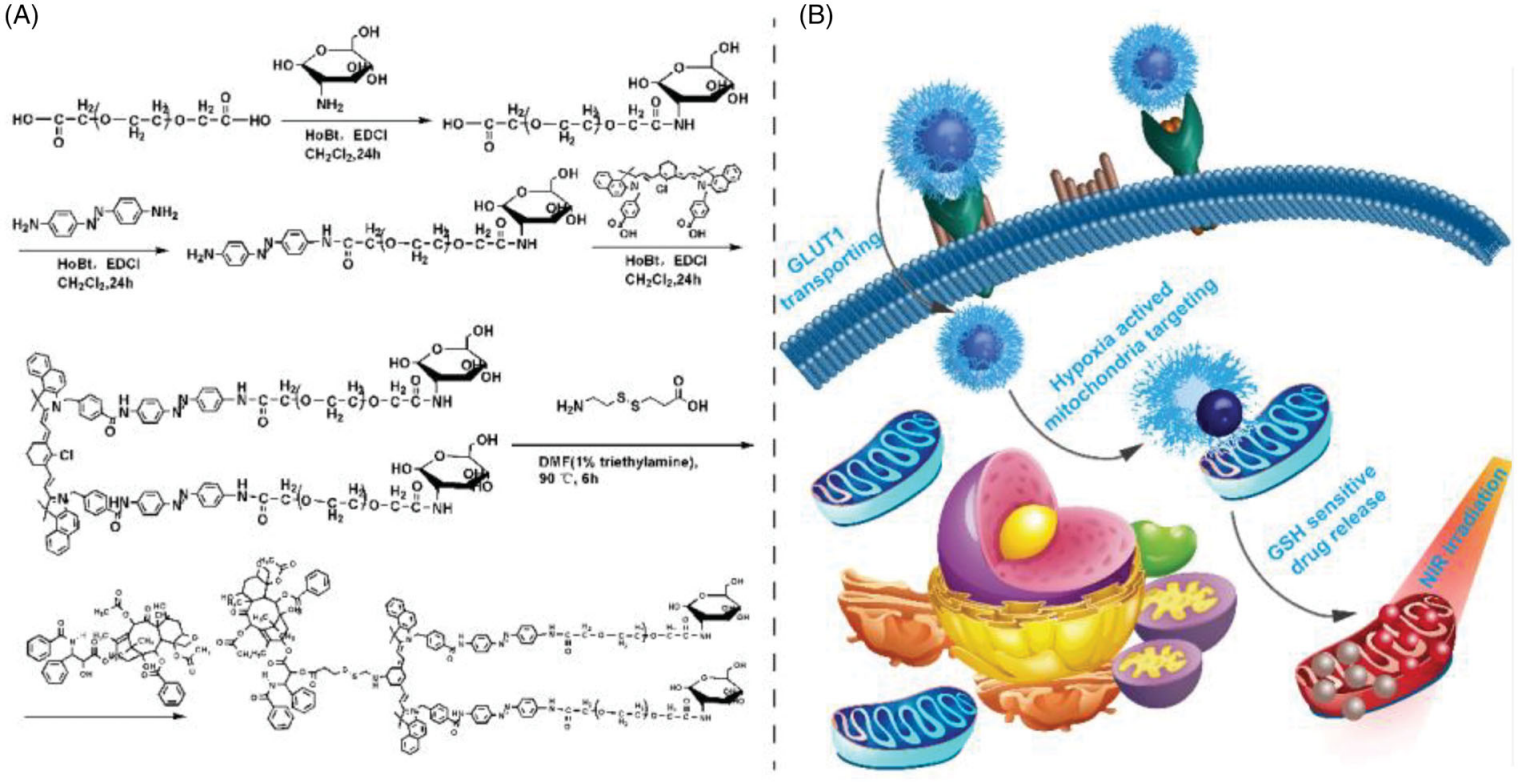
(a) The synthetic process of Glu-PEG-Azo-IR808-S-S-PTX conjugate; (b) its action mechanism after self-assembled into micelle.

Based on this, we constructed a GLUT1 targeting and tumor micro-environment responsive polyprodrug-based micelle for tumor therapy. As shown in Figure 1, the prodrug IR808-S-S-PTX was served as the hydrophobic block and modified with the glycosylated PEG as the hydrophilic shell through a hypoxia sensitive linker p-aminoazobenzene (Azo) to obtain the amphiphilic polyprodrug conjugate glucose-PEG-Azo-IR808-S-S-PTX. When dissolved in water, it self-assembled into nanosized micelle. Following intravenous administration, the micelle could be transported by the over-expressing GLUT1 of tumor cell and hydrophilic PEG shell detached under the hypoxic TME. For the prodrug IR808-S-S-PTX, it ruptured into paclitaxel (PTX) and IR808 under the GSH reductive TME, which acted on the tubulin and mitochondria, respectively. With laser irradiation, the mitochondria were destroyed by photothermal effect, and thus tumor proliferation and metastasis were inhibited.

## 2. Materials and methods

### 2.1. Materials and reagents

Paclitaxel (PTX), p-aminoazobenzene (Azo), and IR808 were purchased from Abmole Bioscience Inc. (Shanghai, China). PEG polymers (MW 1500 Da) were purchased from Jiankai Technology Co., Ltd (Beijing, China). Dulbecco’s modified eagle’s medium (DMEM), fetal bovine serum (FBS), and 3-(4,5-dimethyl-2-thiazolyl)-2,5-diphenyl-2-H-tetrazolium bromide (MTT) were purchased from Gibco Life Technology Company (Grand Island, NY). RIPA lysis buffer and BCA protein assay kit were purchased from Aladdin Co., Ltd (Beijing, China). 3-[(2-Aminoethyl)dithio]propionic Acid (AEDP), Mitotracker green, Hoechst, JC-1 dyeing working solution, ATP assay kit, and other chemical reagents were purchased from Thermo Fisher Scientific Inc. (Waltham, MA. USA). Methanol, methylene chloride (DCM) and other chemical reagents were obtained from Sinopharm Chemical Reagent Co., Ltd (Shanghai, China).

### 2.2. Synthesis of Glu-PEG-Azo-IR808-S-S-PTX conjugate

Glucose-PEG-COOH and excess Azo were dissolved in DCM, added with HOBT and EDCI, and stirred overnight. The reaction solution was purified by silica gel column chromatography with mobile phase DCM/MeOH (50:1). Then, the GLU-PEG-Azo and IR808 were dissolved in DCM, added with HOBT and EDCI, and stirred overnight. The reaction mixture was dialyzed against ultrapure water. Following freeze-drying, the product GLU-PEG-Azo-IR808 was dissolved in DMF containing 1% triethylamine, added with AEDP, stirred for 6 h at 90 °C. The reaction was purified by dialysis. The GLU-PEG-Azo-IR808 and PTX were dissolved in DCM and reacted for 24 h with DMAP and EDCI as catalysts. After evaporating the DCM by reduced pressure distillation, the residue was re-dissolved in water and dialyzed against ultrapure water to obtain the final product GLU-PEG-Azo-IR808-S-S-PTX conjugate. At the same time, the mPEG-Azo-IR808-S-S-PTX and GLU-PEG-Azo-IR808-C-C-PTX conjugates were also synthesized as control. The chemical structures of all conjugates were analyzed using ^1^HNMR, UV-Vis and fluorescence spectra.

The hypoxia sensitivity of conjugates was evaluated using the sodium dithionite as hypoxia activator (Hofstetter et al., 2019). The GLU-PEG-Azo-IR808-S-S-PTX conjugate (1 mg/mL) was incubated with different concentration of sodium dithionite (0.05, 0.075, and 0.1 mg/mL) for 10 min, and then it was incubated with sodium dithionite (0.1 mg/mL) for different time (2, 4, and 6 h). The absorption spectrum of the mixture solution was scanned by an UV-Vis spectrophotometer (Shimadzu UV-3600i Plus, Tokyo, Japan).

### 2.3. Micelle construction and characterization

The dialysis method was employed to prepare micelles (Shen et al., 2020). Briefly, 10 mg conjugates were dissolved in 2.0 mL DMF and dropwisely added into 4.0 mL H_2_O, then the mixture was transferred into a dialysis bag (MWCO 1000) and dialyzed against water for 24 h, and the polymer-drug conjugates-based micelles were obtained by lyophilization. The critical micelle concentration (CMC) was determined using the surface tension method as described elsewhere (Scholz et al., 2018). The particle size and zeta potential of micelles were determined using a DLS at the concentration of 1 mg/mL (Nicomp 380 Zeta Potential/Particle Sizer, Santa Barbara, CA). The morphology of micelles was observed by the TEM (JEM-F200, JEOL, Japan). The photothermal conversion efficiency was determined with an 808 nm laser (laserwave LWIRPD-5F, Beijing, China) at different power density and micelle concentrations, and the temperature was recorded by a platinum resistance thermometer (YOWEX A YET710 YET-710, Shenzhen, China). The drug release profile of micelles was studied using dialysis method. The micelles containing 1 mg paclitaxel were sealed into dialysis bags, immersed into 30 mL PBS containing Tween-80 (0.5%) and GSH (0, 1 or 10 mM), and shaken in a 37 °C water bather at 100 rpm. About 1 mL release medium was sampled at predefined time and the PTX concentration was determined using HPLC at 230 nm with acetonitrile/water (50:50, v/v) as the mobile phase.

### 2.4. Cytotoxicity

The MTT method was used to evaluate the cytotoxicity of micelles. Briefly, A549 cells were seeded in 96-well plates at a concentration of 1 × 10^4^ cells/well and maintained for 24 h at 37 °C. Then, the cells were treated with micelles for 48 h at the concentration gradient of 1.5 × 1 0 ^−3^–1.5 × 10^3^ nM (represented as PTX). About 20 μL MTT (5 mg/mL) was added in each well and incubated with cells for 4 h. Following discarding cell culture medium, 200 μL DMSO was added to dissolve the formazan crystal and the absorbance at 570 nm was recorded using a microplate reader (BioRad, Model 680, Hercules, CA).

### 2.5. Cell apoptosis

Annexin V-FITC/PI double staining was used to detect the apoptosis effect (Ma et al., 2020). A549 cells were planted in 6-well plates with 1 × 10^6^ cells/well. After reaching a confluence of 70–80%, the cells were treated with 3 mL drug solutions at the concentration of 5 nM for 24 h. Then, the A549 cells were washed with pre-cooled PBS and cultured for another 24 h. After that, the cells were digested with trypsin without EDTA, collected, and resuspended in 100 μL of pre-cooled PBS. Then, the cells were stained with 5 μL Annexin V-FITC and 5 μL PI, and incubated in dark for 15 min. Diluting with 400 μL PBS, the fluorescence intensity of cells was measured by a flow cytometry (CytoFLEX, Suzhou, China).

### 2.6. Cell metastasis

Both wound scratch assay and transwell migration assay were utilized to evaluate anti-metastasis effect of micelles (Lee et al., 2014). For wound scratch assay, cells were seeded in 12-well plates for 24 h at 37 °C. The cell monolayer was scratched using a 200 μL pipette tip and washed twice with fresh PBS. Micelles were added into each well at a concentration of 10 μg/mL and co-incubated with cells for 24 h at 37 °C. Discarding culture media, the scratched area was observed using an optical microscope. For transwell migration assay, cells were dispersed in FBS-free culture media and seeded onto the upper surface of transwell chamber at a density of 1 × 10^5^ cells/mL, and the outside chamber was filled with 600 μL complete medium as chemoattractant. Following co-incubated with micelles for 12 h, the cells on the bottom of upper chamber were immobilized and stained with 0.1% crystal violet. The cells were observed by an optical microscope and the absorbance at 470 nm was also determined.

### 2.7. Mitochondria targeting

The mitochondria targeting ability of micelles was conducted under both hypoxic and normoxic environments. The A549 cells were seeded in 24-well plates at a concentration of 1 × 10^5^ cells/mL for 24 h at 37 °C. Micelles were added into each well at a concentration of 5 μg/mL and co-incubated with cells for 12 h at 37 °C. The hypoxia-activated mitochondria targeting was evaluated using the AnaeroPack as described before (Wen et al., 2020). The drug solution was replaced with fresh cell culture media, and then the nucleus and mitochondria were stained with Hoechst 33342 and Mito-Tracker Green, respectively. The colocalization of micelles was observed using a laser scanning confocal microscope (LSCM, Olympus FV1000, Tokyo, Japan).

### 2.8. *In vivo* fluorescence imaging

The A549 tumor-bearing animal model was established as described before (Ehlerding et al., 2019). Briefly, A549 cells were inoculated into the right axillary of nude mice. When the tumor grew to 100 mm^3^, the mice were randomly divided into five groups and administrated with micelles at a concentration of 5 mg/kg (represented as PTX). The biodistribution of micelles were observed using an *in vivo* imaging system (CRi Maestro, MA, USA) at predefined time 0.5 h, 1 h, 2 h, 4 h, 8 h, and 12 h. After 12 h, the organs (heart, liver, spleen, lung, kidney and tumor) were harvested for observation. Besides, frozen sections of tumor tissues were prepared, the nucleus and mitochondria were stained with Hoechst 33342 and Mito-Tracker Green, respectively. The distribution of micelles in mitochondria was observed using the LSCM. All animal studies were performed according to the Guide for the Care and Use of Laboratory Animals (China), and the Animal Care and Use Committee of Beijing University of Chinese Medicine approved the animal study protocols.

### 2.9. Anti-tumor efficacy and safety

The tumor-bearing mice were randomly divided into four groups and administrated with micelles via tail vein injection at a dose of 5 mg/kg every 2 days for 16 days. The body weight and tumor volume were recorded every two days. At the last day, the mice were sacrificed by pick of eyeball and blood was collected for routine biochemistry assays (ALT, AST, BUN, and CER).

### 2.10. ATP content and mitochondria membrane potential assay

For mitochondria membrane potential (MPP) assay, the cells were treated with micelles under different conditions and then co-incubated with 1 mL JC-10 staining solution for 20 min at 37 °C. Following washed with JC-10 staining buffer, the cells were observed using the LSCM. For ATP content assay, the A549 cells in logarithmic growth phase were treated with micelles for 24 h under hypoxic/normoxic or ±808 nm laser conditions. After trypsinization, the cells were collected at 1500 r/min, added with 1 mL extracting solution, and subjected to ultrasonication for 1 min. Following centrifugation at 10,000 rpm for 10 min, the supernatant was collected, added with 0.5 mL chloroform, and centrifugated at 10,000 rpm for 3 min. The supernatant was placed on ice and processed with the specification of ATP content determination kit.

### 2.11. Statistical analysis

All data were expressed as mean ± SD (*n* = 6). Data were processed with SPSS statistical software (SPSS, Chicago, IL). Statistically significant differences between groups were determined with Student’s *t*-test. When *p* < 0.05, it was considered as statistically significant.

## 3. Results and discussion

### 3.1. Characterization of conjugates

**Figure 2.**
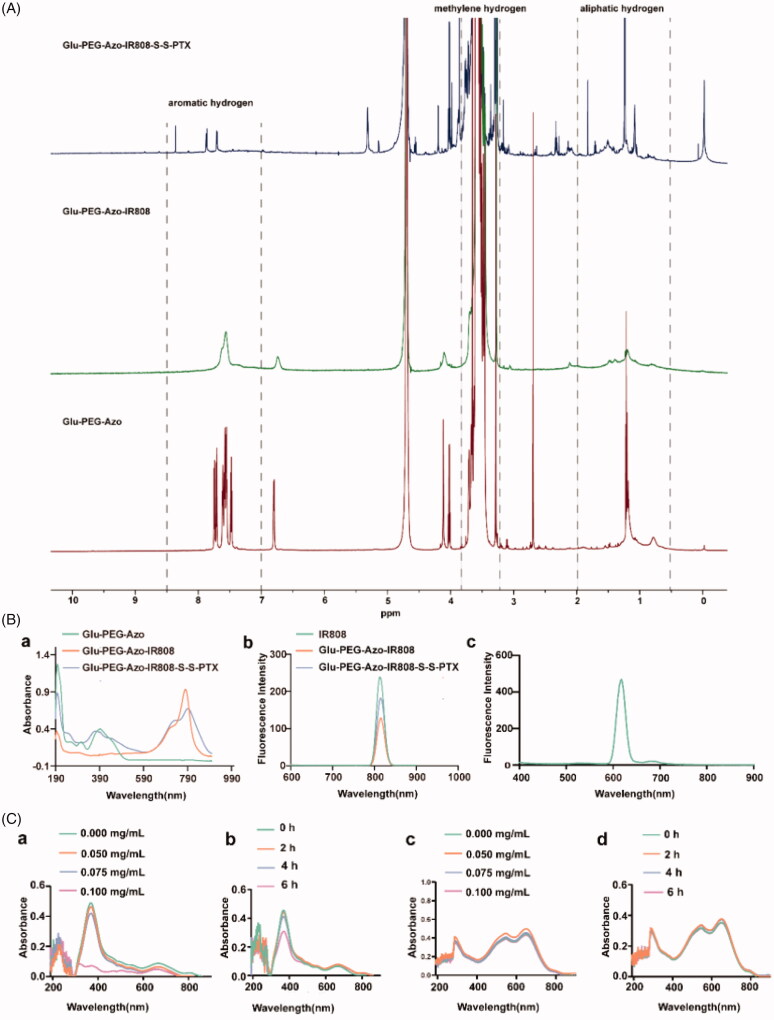
Characterization of the Glu-PEG-Azo-IR808-S-S-PTX conjugate. (A) ^1^HNMR of conjugates; (B) UV-Vis absorption spectra of conjugates (a) and fluorescence emission spectra of the conjugates excited at 660 nm (b, c); (C) UV-Vis absorption spectra change of the Glu-PEG-Azo-IR808-S-S-PTX conjugate (a, b) and Glu-PEG-IR808-S-S-PTX conjugate (c, d) following treatment with Na_2_S_2_O_4_ at different concentrations and time.

The glycosylated derivative of PEG (glucose-PEG) and IR808 were firstly conjugated by p-aminoazobenzene via amidation reaction (Glu-PEG-Azo), and the chemical structure was confirmed by ^1^HNMR spectra. As shown in Figure 2(a), the peaks at δ (ppm) 0.5–2.0 could be assigned to aliphatic protons of glucose, and the peak at δ (ppm) 3.6 was the characteristic peak of methylene, which could be assigned to PEG. Then, the IR808 also conjugated with Azo via amidation reaction (Glu-PEG-Azo-IR808). Following the AEDP nucleophilic substitution of the chloride of IR808, PTX was conjugated with AEDP via esterification reaction. The peaks at δ (ppm) 7.0–8.0 were the characteristic peaks of phenyl groups belonging to Azo and IR808. The chemical structures were also confirmed by UV-Vis and fluorescence spectra. As shown in Figure 2(b), the characteristic peaks of Azo and IR808 could be found at 365 nm, 495 nm, and 808 nm, respectively. Besides, the peak intensity at 808 nm decreased after the chlorine atom of IR808 was substituted by the AEDP. And the emission ray peak of IR808, Glu-PEG-Azo-IR808, and Glu-PEG-Azo-IR808-S-S-PTX was at wavelength of 808 nm when excited by 660 nm, whereas the peak intensity at 808 nm decreased and a new peak at 620 nm appeared when the chlorine atom of IR808 was substituted by the AEDP. These results could demonstrate that the Glu-PEG-Azo-IR808-S-S-PTX conjugate was successfully synthesized. Furthermore, the hypoxia-sensitivity of the Glu-PEG-Azo-IR808-S-S-PTX conjugate was evaluated with sodium dithionite (Na_2_S_2_O_4_) as an exogenous generator of hypoxia. When the incubation concentration and time increased, the characteristic peak of Azo (365 nm) decreased, while the characteristic peak of benzene (220 nm) increased (Figure 2(c)), which was due to the Azo bond breaking. However, for the Azo non-containing conjugate, its spectra had no change after treatment with Na_2_S_2_O_4._ So, the hypoxia sensitivity of the Glu-PEG-Azo-IR808-S-S-PTX conjugate was concentration and time dependent.

### 3.2. Characterization of conjugate-based micelle

**Figure 3.**
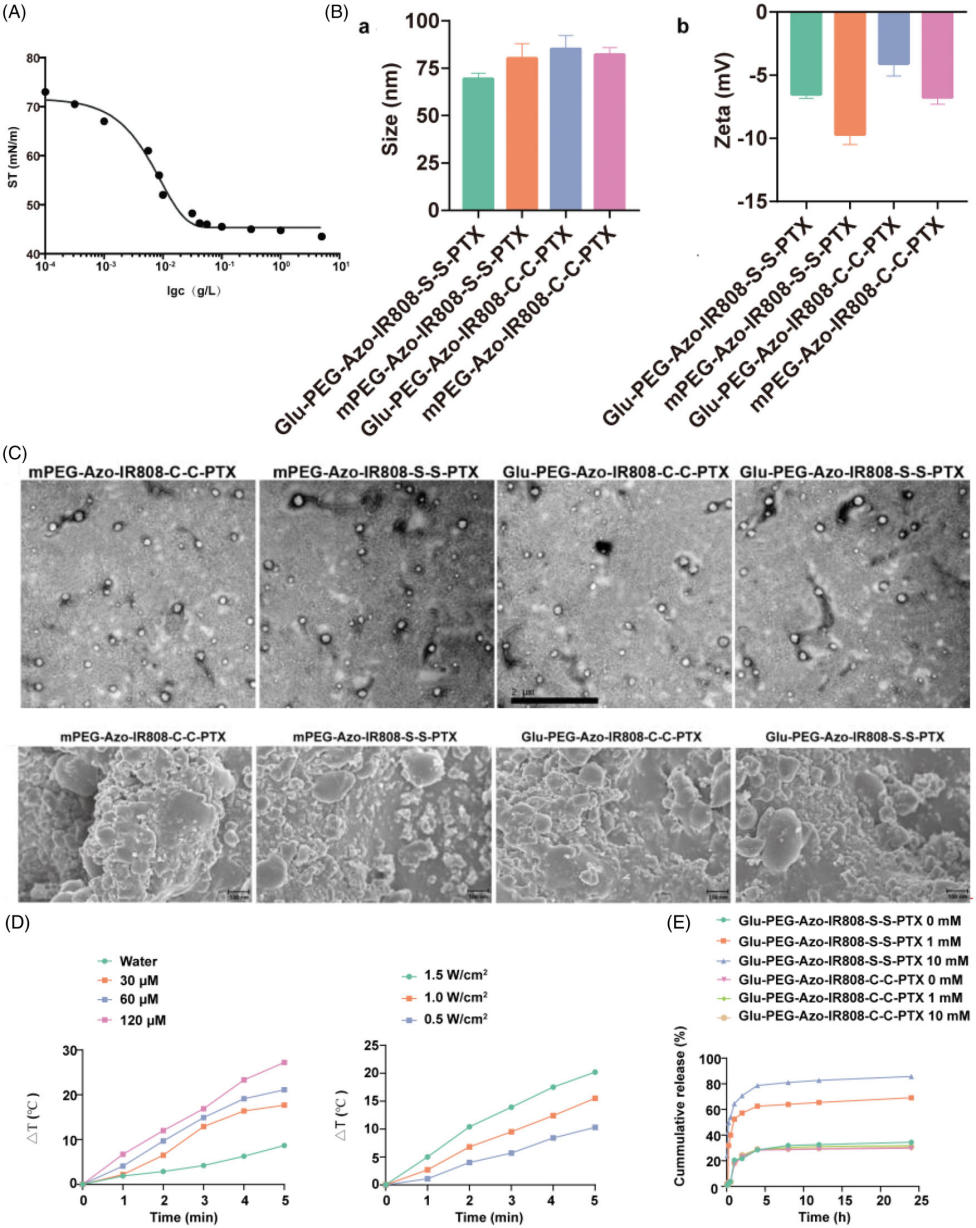
Characterization of conjugate-based micelles. (a) Surface tension at different concentration of Glu-PEG-Azo-IR808-S-S-PTX conjugate; (b) Particle size and zeta potential of micelle determined by dynamic light scattering; (c) Morphology of micelles observed by TEM; (d) Concentration and power density dependent photo-thermal conversion efficiency when irradiation with an 808 nm laser; (e) *In vitro* drug release of micelles at different concentrations of GSH. The scale bar was 2 μm.

The amphiphilic conjugates tend to self-assemble into micelles when dissolved in water. As shown in Figure 3(a), the critical micelle concentration (CMC) of the Glu-PEG-Azo-IR808-S-S-PTX conjugate was about 5.0 × 10^−3 ^nM, which indicated that it could easily assemble into micelle. As the micelle was composed of conjugates, its drug loading could be calculated by the chemical formula of conjugates, and the drug loading of the Glu-PEG-Azo-IR808-S-S-PTX micells calculated to be about 19%. The particle size of micelles ranged from 50–100 nm, and the zeta potential was negatively charged (Figure 3(b)), which was beneficial for its *in vivo* long circulation (Suchaoin et al., 2017). The morphology was further confirmed by the TEM, and the micelles were approximate to sphere particles with particle size about 100 nm (Figure 3(c)). The photothermal conversion efficiency was both concentration dependent and irradiation dependent. At the highest concentration, the temperature increased from 20 °C to 50 °C (Δt 30 °C) within 5 min (Figure 3(d)), which was requisite for ablating the tumor (Liu et al., 2020b). The *in vitro* drug release of disulfide bond containing micelle was more quick than the control micelle, and the drug release speed was GSH concentration dependent (Figure 3(e)). So, the micelle could quickly release drug after entering into tumor cell and avoiding drug release in normal cells.

### 3.3. *In vitro* pharmacodynamics

#### 3.3.1. Cytotoxicity

The anti-tumor potential of micelles was firstly evaluated by the cytotoxicity. As shown in Figure 4(a), the Glu-PEG-Azo-IR808-S-S-PTX micelle showed stronger inhibition ability than the mPEG-Azo-IR808-S-S-PTX micelle, indicating the glucose modification could promote cellular uptake thus enhancing the cellular drug concentration. Under hypoxic environment, the cytotoxicity of micelles significantly increased and had higher cytotoxicity compared with that under normoxic environment. The possible reason might be due to the IR808-S-S-PTX conjugate which would release and act on tumor cells when the PEG detached from PAMAM under hypoxia condition. Due to the positive charge of IR808, the IR808-S-S-PTX conjugate was more easily adsorbed and endocytosed by the cell, which induced higher cytotoxicity. When irradiation with 808 nm laser, the cytotoxicity of micelles further enhanced, the IC50 of the Glu-PEG-Azo-IR808-S-S-PTX micelle decreased by 7 folders reaching to 1.30 nM. Thus, the photothermal effect induced by IR808 could strengthen the chemotherapeutic effect of PTX.

Figure 4.Pharmacodynamics evaluation on the A549 cells. (a) Cytotoxicity of micelles on A549 cells; (b) Flow cytometric analyses of A549 cells using annexin V-FITC/PI dual staining after treatment with micelles; (c) Microscopic images and quantitative analysis of the wound-healing after treatment with micelles; (d) Microscopic images and quantitative analysis of the transwell migration after treatment with micelles. *p < 0.05.

**Figure:**
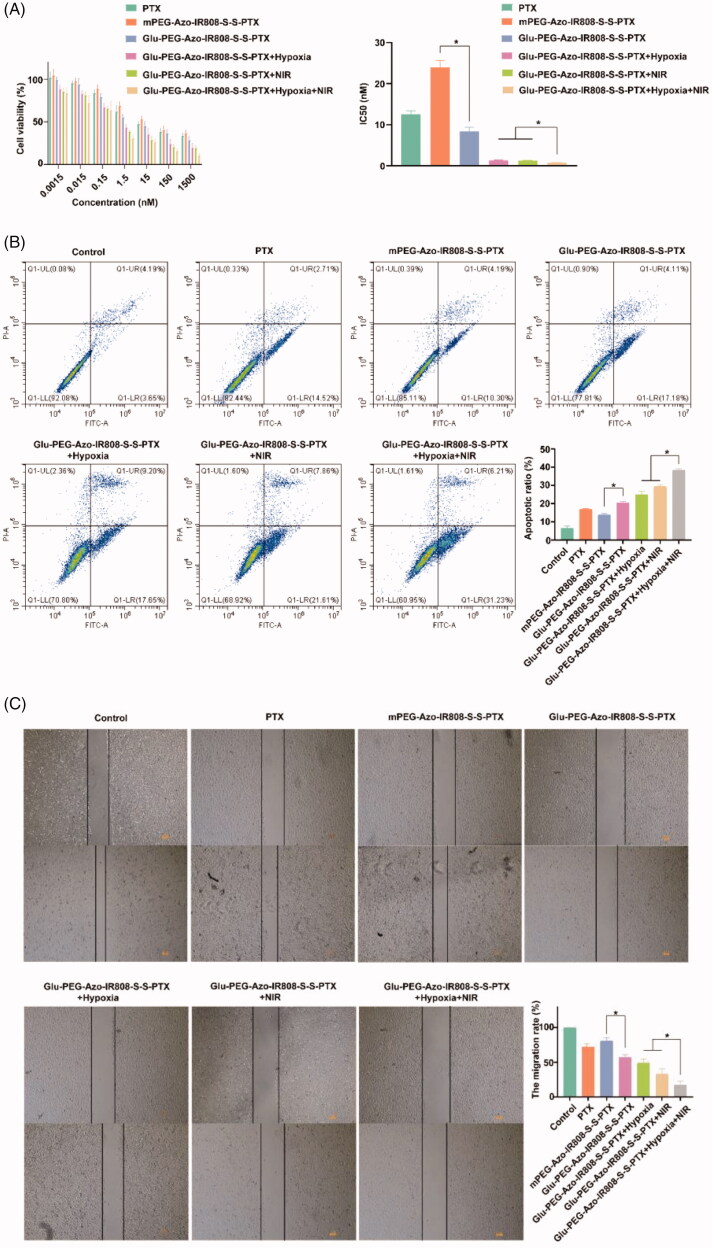

**Figure:**
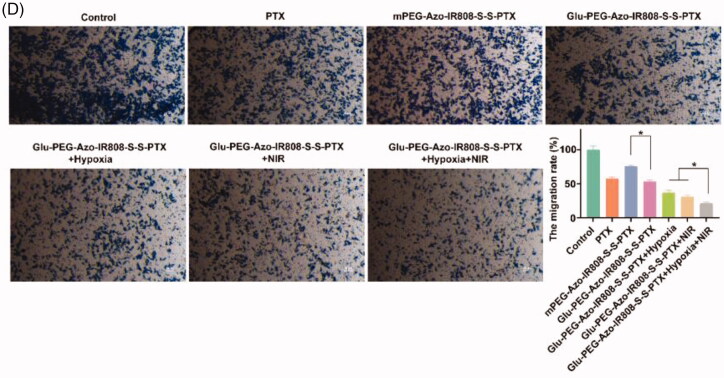

#### 3.3.2. Cell apoptosis

The anti-tumor potential of micelles was then assessed by cell apoptosis. As shown in Figure 4(b), all administration groups had pro-apoptoxic effect. For the Glu-PEG-Azo-IR808-S-S-PTX micelle, its cell apoptotic ratio increased after treatment with laser irradiation both under normoxic and hypoxic conditions (*p* < 0.05), which demonstrated that the phtotothermal effect could induce cell apoptosis. Especially under hypoxia condition, the apoptotic ratio increased from 17.65% to 31.23%, and the result might be due to the mitochondria which was more sensitive to thermal under hypoxia condition (Tan et al., 2019).

#### 3.3.3. Cell metastasis

The inhibition of cell metastasis was evaluated by wound healing and transwell migration. As shown in Figure 4(c), the area of scratch for the control group almost completely recovered after 12 h, whereas for micelle groups, they exhibited migration inhibition effect with varying degrees. The migration rate of Glu-PEG-Azo-IR808-S-S-PTX micelle group was 57.0%. After irradiation with laser, the migration rate further decreased to 32.89%, indicating the synergistic effect of PTX and IR808. Likewise, compared with control group, the number of cells for micelle groups that migrated through the membrane was less (Figure 4(d)). These results demonstrated that the Glu-PEG-Azo-IR808-S-S-PTX micelle could efficiently inhibit the metastasis of tumor cells.

### 3.4. Mitochondria targeting evaluation on cells

**Figure 5.**
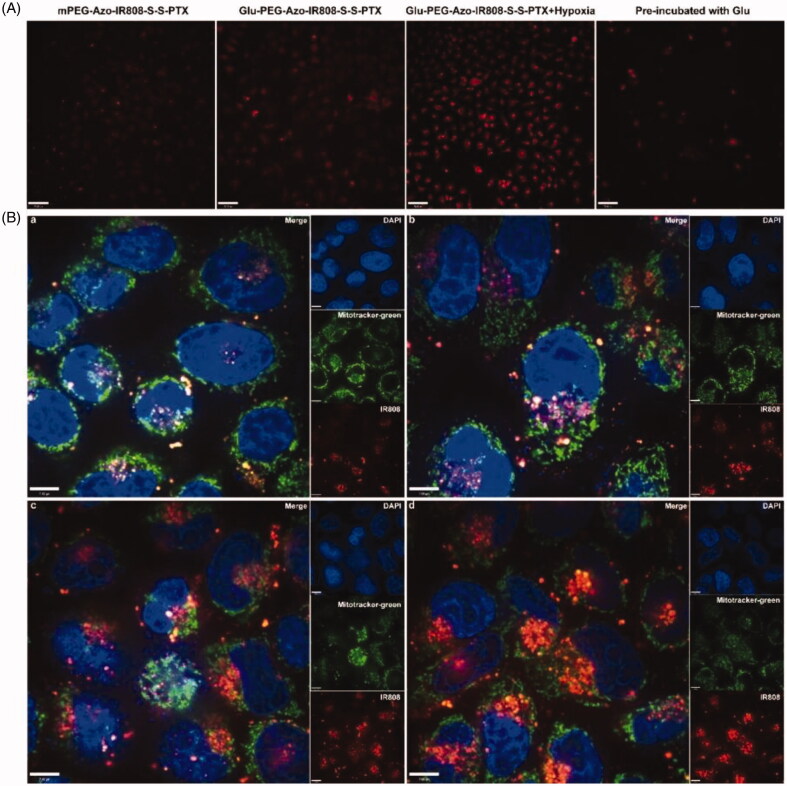
Cellular uptake and subcellular localization evaluation. (a) LSCM images of A549 cells after co-incubation with micelles, the scale bar was 70 µm; (b) LSCM images of A549 cells after treating with mPEG-Azo-IR808-S-S-PTX and Glu-PEG-Azo-IR808-S-S-PTX under normoxic conditions (a, b) and hypoxic conditions (c, d), the scale bar was 7 µm.

Due to the fluorescence of IR808, the cellular uptake of micelles could be observed directly without modifying with other fluorescent reagents. As shown in Figure 5(a), under normoxic environment, the fluorescence intensity of the Glu-PEG-Azo-IR808-S-S-PTX micelle was much higher than the mPEG-Azo-IR808-S-S-PTX micelle group, and it could be blocked by pre-treating with glucose, which demonstrated that the cellular uptake was mediated by GLUT1. Furthermore, the co-localization experiments confirmed that the Glu-PEG-Azo-IR808-S-S-PTX exhibited stronger cellular uptake than the mPEG-Azo-IR808-S-S-PTX micelle under normoxic environment (Figure 5(b)). And they tend to target the nucleus. Although under hypoxic environment, the red fluorescence of IR808 co-localized with the green fluorescence of mitochondria quite well, which further demonstrated that the IR808 could effectively targeting the to the mitochondria following the Azo and disulfide bond got broken.

### 3.5. *In vivo* tumor targeting evaluation

**Figure 6.**
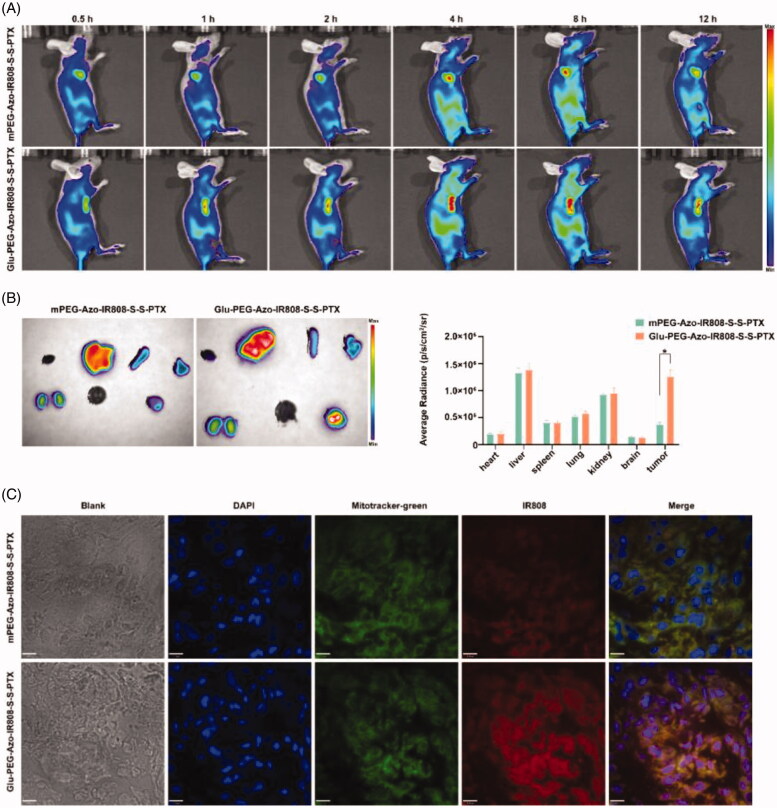
Tumor and mitochondria targeting evaluation on A549 tumor-bearing mice. (a) Fluorescence imaging of A549 tumor-bearing nude mice following administration with micelles; (b) *Ex vivo* fluorescence imaging of major organs and quantitative analysis of their fluorescence intensity; (c) LCSM images of tumor frozen sections. The scale bar was 11.00 μm, *p < 0.05.

The *in vivo* tumor targeting was evaluated through observing the distribution of micelles in the tumor-bearing nude mice. As shown in Figure 6(a), all micelles rapidly distributed over the whole body after intravenous injection. The Glu-PEG-Azo-IR808-S-S-PTX micelle began to accumulate at tumor for 2 h and last to 12 h. Meanwhile, the fluorescence intensity of the Glu-PEG-Azo-IR808-S-S-PTX micelle was much higher than the mPEG-Azo-IR808-S-S-PTX micelle. The *in vitro* tissue observation further confirmed that the fluorescence intensity of the Glu-PEG-Azo-IR808-S-S-PTX micelle was 5-fold compared to the mPEG-Azo-IR808-S-S-PTX micelle (Figure 6(b)). Then, the mitochondria-targeting evaluation was conducted by preparing cryostat sections of tumors. As shown in Figure 6(c), the red fluorescence of the Glu-PEG-Azo-IR808-S-S-PTX micelle was stronger than the mPEG-Azo-IR808-S-S-PTX micelle and colocalized well with the green fluorescence of the mitochondria. These results demonstrated that the Glu-PEG-Azo-IR808-S-S-PTX micelle could target both tumor and its mitochondria.

### 3.6. Anti-tumor efficacy on animals

**Figure 7.**
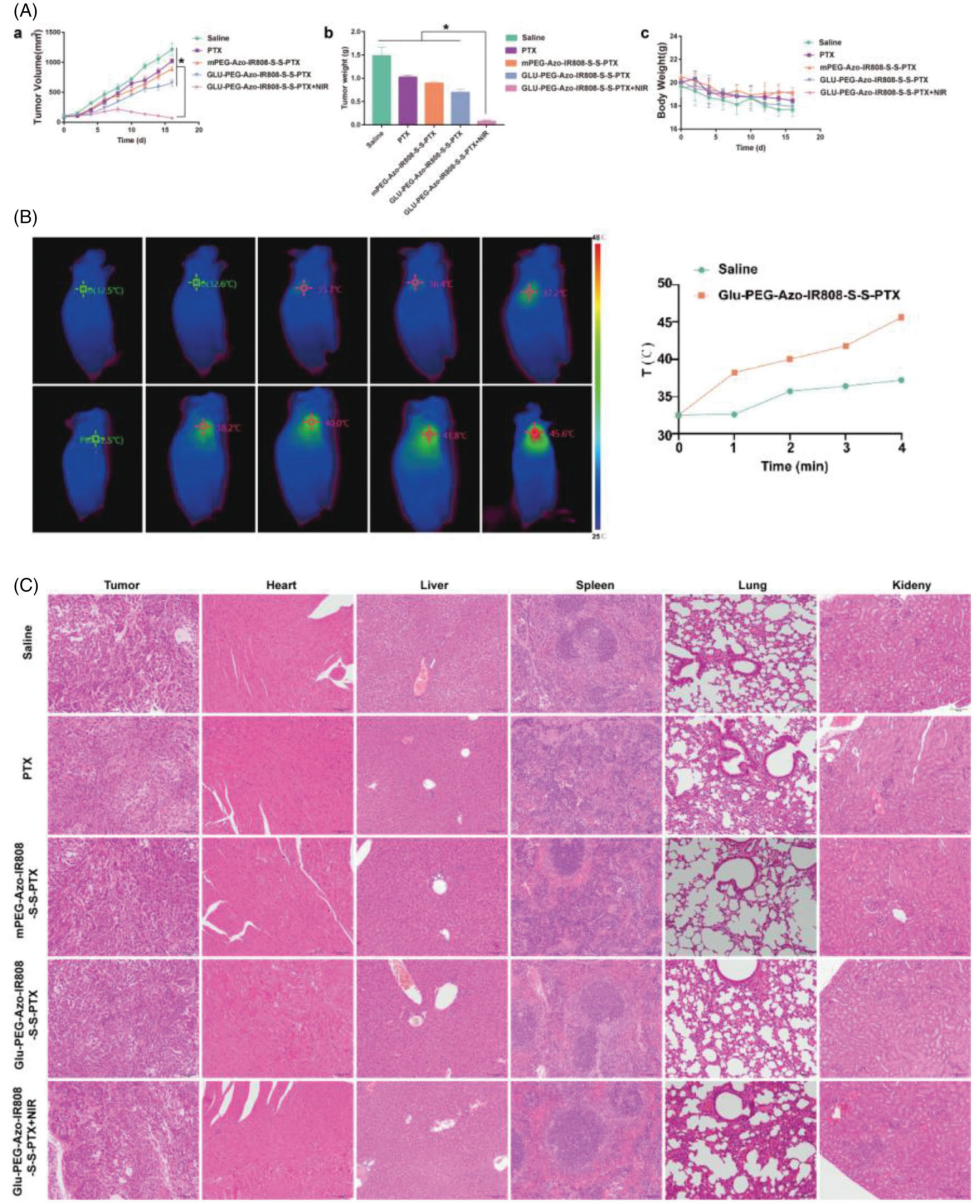
Anti-tumor efficacy of micelles on the A549 tumor-bearing nude mice. (a) Change of tumor volume following treatment with micelles (a), Weight of tumors excised from mice at day 16 (b); Body weight changes during the treatment period (c); (b) The temperature change profiles at the tumor sites under laser irradiation (1.5 W/cm^2^, 4 min); (c) H&E staining of major tissue sections. The scale bar was 100 μm. *p ＜ 0.05.

*In vivo* therapeutic efficacy was evaluated on the A549 tumor-bearing nude mice. As shown in Figure 7(a), the tumor volume of administration groups was less than the saline group, which indicated that the tumor growth was inhibited after drug administration. Meanwhile, compared with the PTX group, all micelle groups exhibited obvious higher tumor inhibition efficacy. The Glu-PEG-Azo-IR808-S-S-PTX micelle showed superior anti-tumor ability compared with the mPEG-Azo-IR808-S-S-PTX micelle that might be due to its better tumor-targeting ability. Interestingly, when the Glu-PEG-Azo-IR808-S-S-PTX micelle irradiated by laser, the tumor almost disappeared. Moreover, there was no obvious body weights loss in mice injected with different formulations. The temperature of tumor was higher than 45 °C following irradiation with 808 nm laser at 1.5 W/cm^2^ for 4 min (Figure 7(b)), indicating the photothermal effect could efficiently ablate the tumor. To further investigate the tumor metastasis and *in vivo* toxicity, the major organs of the treated mice were sliced and stained by H&E staining for histology analysis (Figure 7(c)). For micelle groups, no tumor metastasis forci could be observed, whereas there were obvious metastatic adenocarcinomas in lung or spleen tissues for the PTX and saline groups. And there was no pathological variation on the H&E-stained sections of the main organs in all micelle groups. These results suggested that no apparent systemic or tissue toxicity was induced by these micelles in the treated animals. The low toxicity of micelles could be attributed to their tumor-specific bio-distribution and reduced drug release in the normal tissues.

### 3.7. Anti-tumor mechanism on cells

**Figure 8.**
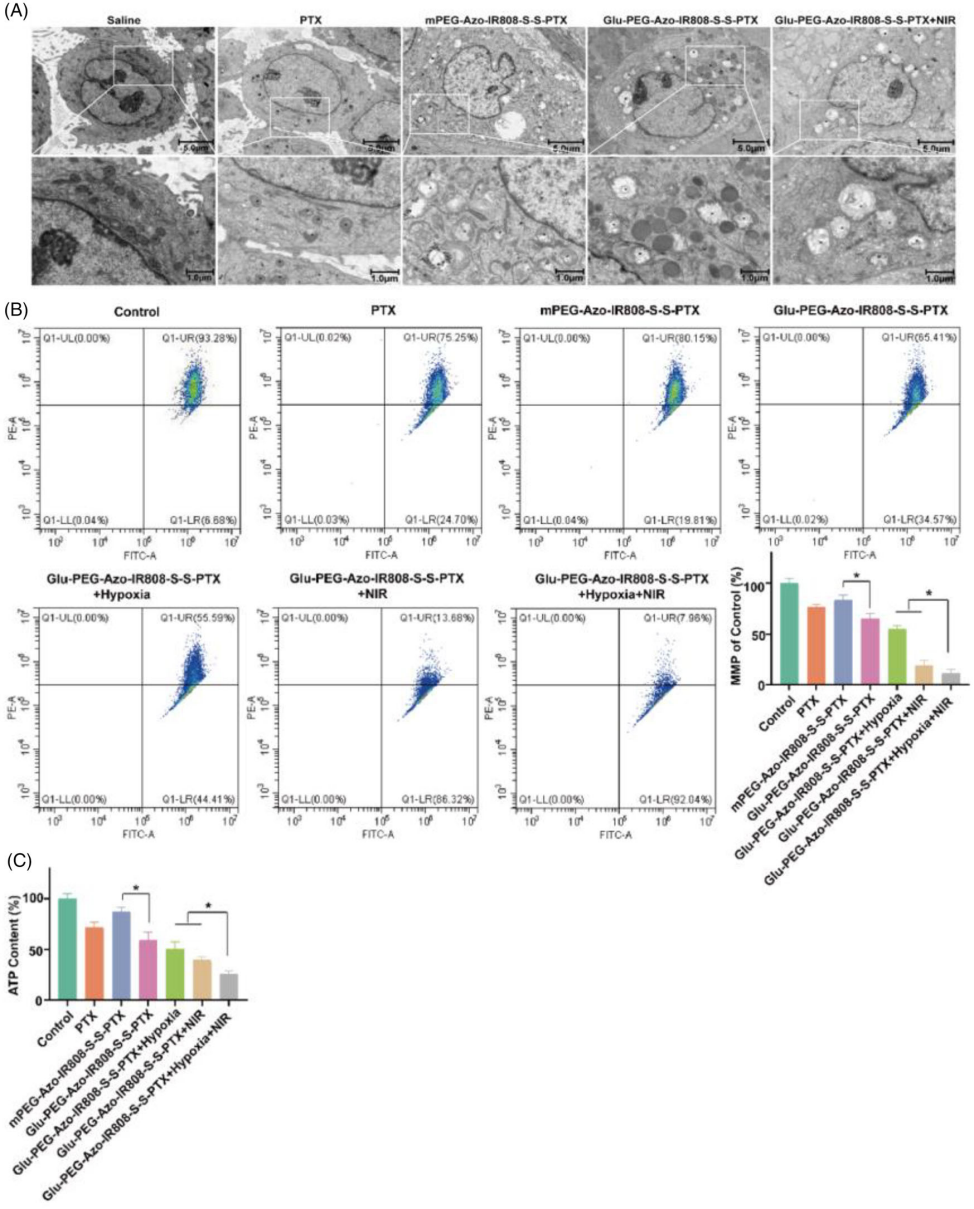
Mechanism of anti-tumor growth and metastasis through acting on the mitochondria. (a) Mitochondria ultrastructure observed by TEM; (b) Flow cytometric analyses of A549 cells staining with JC-1 probe after different treatments as indicated; (c) ATP content of cells after different treatments as indicated. * *p* ＜ 0.05. M: mitochondria.

The potential antitumor mechanism of micelles was illustrated by exploring its effect on cell mitochondria. The TEM observation of tumor cell mitochondria demonstrated that the mitochondria were seriously destroyed by the Glu-PEG-Azo-IR808-S-S-PTX micelle (Figure 8(a)). The mitochondria were swollen and expanded, and the crista disappeared. After treatment with NIR laser irradiation, some mitochondria appeared vacuolation with a large number of microfilaments and microtubules. Besides, the MMP of Glu-PEG-Azo-IR808-S-S-PTX micelle groups was significantly lower than the PTX group, indicating that the IR808 could induce mitochondrial depolarization after targeting to mitochondria (Figure 8(b)). The similar changing trend also could be found in the ATP content (Figure 8(c)), further indicating that the micelle could disturb the mitochondrial function and reduce intracellular ATP production. It is noteworthy that both the MMP and ATP content extremely decreased following irradiation with laser, which illustrated that the photothermal effect had stronger effect on mitochondria compared with the chemotherapeutic effect.

## 4. Conclusion

In this study, we fabricated a GLUT1 targeting, hypoxia, and GSH responsive polymer-drug conjugate-based micelle to combine chemotherapy and photo-thermal therapy. The micelle not only could be specifically transported by the GLUT1 of tumor cells, but also efficiently delivered PTX and IR808 to the tubulin and mitochondria under tumor microenvironment, ultimately leading to cell apoptosis through destroying mitochondria and depleting ATP production. *In vivo* assays also revealed that the micelle mainly accumulated in tumor tissues and its mitochondria exhibited super high suppression efficiency on tumor growth and metastasis as well as no serious toxic effects toward the whole body. Therefore, this work provided a concise and promising nanomedicine for tumor therapy.
